# Perceptions of foot health services from the perspective of patients with rheumatoid arthritis in Finland

**DOI:** 10.1002/jfa2.12004

**Published:** 2024-04-03

**Authors:** Minna Stolt, Sasu Hyytiä, Riitta Suhonen

**Affiliations:** ^1^ Department of Nursing Science University of Turku Turku Finland; ^2^ Wellbeing Services County of Satakunta Pori Finland; ^3^ Turku University Hospital Wellbeing Services County of Southwest Finland Turku Finland

**Keywords:** foot health, foot health services, perceptions, qualitative study, rheumatoid arthritis

## Abstract

**Background:**

Foot health services for people with rheumatoid arthritis (RA) are an important part of their comprehensive care. However, little is known about the perceptions of people with RA have about foot health services. This study aimed to explore how people with RA perceive foot health services.

**Methods:**

A descriptive cross‐sectional survey design was applied. The electronic survey data were collected in April 2023 from people with RA through a national patients' association (*N* = 2400, response rate 24%, *n* = 565). The statistical data were analysed using descriptive statistics and textual data with thematic analysis.

**Results:**

Most of the respondents (*n* = 322, 59%) had used foot health services provided by chiropodist or podiatrist. Those who had used services were mostly satisfied but considered patient education about foot health insufficient. One third reported no visits to foot health services at all because of personal and health service system‐related factors.

**Conclusions:**

Those people with RA who have access to foot health services value and appreciate the services. However, many people with RA do not use foot health services because they perceive availability of such services limited and thus unequal and hard to access. There is a need to develop foot health services for people with RA so that they are easy to access, correspond to their foot health needs and have seamless care paths at different levels of the health care system.

## 1 BACKGROUND

Foot health services for people with rheumatoid arthritis (RA) are an important part of their comprehensive care. RA is the most common type of inflammatory arthritis and globally it affects 460 people per 100,000 of the population [1]. A wide range of foot problems are known to be common in RA. Foot problems are painful and cause significant restrictions to people's daily life. For people with RA, foot problems may hinder their domestic life, leisure activities and have an influence on their working capacity [2]. Due to the progressive nature of RA, all possible efforts should be targeted to individuals' foot health. The prevention of foot problems or ensuring the treatment of already existing foot problems in people with RA are important in maintaining a person's functional health. Therefore, regular monitoring of foot health and access to foot care and podiatry should be the highest priority in the care of people with RA.

Standards of care guidelines for people with inflammatory arthritis recommend that patients with early RA should be referred to podiatry for assessment, advice and intervention [3]. At a later stage, regular foot health checks for people with RA are recommended to monitor the status of foot health and to provide patient education to prevent or care for foot problems [4]. Despite the importance of foot health services, foot health management does not correspond with patients' foot health needs [5, 6]. People with RA are of the opinion that their feet are ignored during routine rheumatology consultations [5, 6, 7, 8]. In their view, the frequency of foot examinations is limited [7] and they are often required to demand that health care professionals examine their feet [9, 10]. However, patients who had accessed foot care services prioritised their foot problems as an important health care need.

People with RA are willing to care for their own feet [9]. However, they are unsure about appropriate care activities, and they need foot health information [9, 11]. To support their foot self‐care, they appreciate the experience of professionals and wish to have patient education on their foot health [7]. People with RA are often unaware of foot health services and they report a lack of knowledge on how to access foot health services [8]. Regarding the access to foot health services, it is reported that referrals to podiatric care is often difficult due to the lack of podiatrists as a resource as well as health care professionals' limited knowledge on the benefits of podiatric care to people with RA [12, 13].

The most common reasons for people with RA to seek foot health services are foot pain, skin and nail lesions and footwear and orthotic needs [14]. However, there seems to be a discrepancy between foot health needs of people with RA and the care provided [15]. Many patients who had visited foot health services expressed dissatisfaction with the care they had received and also commented on a perceived lack of specific RA foot management expertise amongst the podiatry work force [11]. The high costs of foot health services and lack of local referrals to foot health services are often reported as factors limiting access to foot care [8, 9].

Given the importance of foot health services among people with RA, previous studies [8, 9, 11, 14] have reported their experiences of using foot health services. However, little is known about the perceptions of people with RA who do not use foot health services and their views on how foot health services could be developed to meet the foot health needs of people with RA. With this information, foot health services could be developed to respond to the individual foot health needs of people with RA. Moreover, the information could be used to develop the quality of foot health services.

The aim of this study was to explore how people with RA perceive foot health services provided by chiropodist or podiatrist. The following research questions were asked:

‐ Do people with RA use foot health services provided by chiropodist or podiatrist?
  • If yes, how satisfied are they with foot health services?
  • If no, why do they not use foot health services?
‐ How should foot health services for people with RA be developed?

## 2 METHODS

A descriptive cross‐sectional survey design was applied.

### 2.1 Setting

In Finland foot health services in general are delivered by chiropodists (education 2.5 years) or podiatrists (education 3.5 years) in public health care or in private clinics. The chiropodists or podiatrists in public health care work commonly in university hospitals or regional hospitals. There are approximately 100 vacancies for chiropodists or podiatrists in the Finnish public health care that serves 5.5 million inhabitants. The get access to public podiatric care a referral is needed. Public podiatry or foot care is covered by taxation, and clients need to pay only a small appointment fee for the services. Custom‐made footwear, insoles and other personal devices may be received without any costs to clients if a physician recommends that a client would benefit from an individually tailored therapeutic assistive device. In Finland, many podiatrists work as private practitioners, offering their services to all and charging for the service. Private foot health services are not reimbursed, and all costs are payable by the client. Some private foot health practitioners work as subcontractors for hospitals, and through such contracts the foot care and podiatry costs to clients is only a small appointment fee. Regarding foot health care for patients with RA, the current care guideline in Finland [16] recommends that the patient should be referred to podiatry when needed. However, the guideline does not take a position on the frequency of regular foot health assessment or care.

### 2.2 Data collection

The data for this study were collected in April 2023 electronically from people with RA through a national patients' association. An electronic survey was sent to all members of the national patients' association who have registered themselves as having RA (*N* = 2400). One reminder email after 2 weeks was sent. The response rate was 24% (*n* = 565).

The survey, developed for the purposes of this study, consisted of structured and open‐ended questions about perceptions of foot health services (6 questions) and background questions (7 questions, Tables 1 and 2). Regarding perceptions of foot health services, the respondents were asked to report if they have used foot health services provided by the chiropodist or podiatrist for their foot problems (yes/no). Then the frequency of receiving foot health services (once a month, once in 3 months, once in 6 months, once a year, rarely and I do not visit podiatrists at all) was asked. Respondents were also asked how satisfied they were with those foot health services (1 = very satisfied, 2 = satisfied, 3 = not satisfied nor dissatisfied, 4 = dissatisfied, 5 = very dissatisfied). Regarding their foot health needs, the respondents were asked if they have received enough patient education about foot health delivered by health care professionals (yes/no). If respondents were not using foot health services, they were asked with open‐ended questions to describe their reasons and grounds for this decision. In addition, as open‐ended questions, the respondents were asked to describe how they would develop foot health services targeted to people with RA.

**TABLE 1:** Background characteristics of the respondents (*n* = 565).

Variable	Mean (range)	*f* (%)
Age (years)	60.2 (22–87)	
Duration of RA (years)	14.5 (0–70)	
Gender		
Female		491 (90)
Male		57 (10)
Highest level of education		
Elementary school		23 (4)
Primary school		37 (7)
High school		21 (4)
School‐level vocational training		147 (27)
Polytechnic degree		221 (40)
University degree		102 (19)
Importance of foot health		
Very important		463 (84)
Important		84 (15)
Somewhat important		6 (1)
Effect of foot health on daily activities		
Very much		230 (42)
A lot		213 (39)
A lot or less		64 (12)
A little		23 (4)
Very little		21 (4)
Self‐evaluated level of foot health	6.7 (0–10), median 7	
1		4 (1)
2		9 (2)
3		20 (4)
4		38 (7)
5		69 (13)
6		79 (14)
7		132 (24)
8		120 (22)
9		68 (12)
10		13 (2)

**TABLE 2:** Frequency and satisfaction with foot health care services and foot‐related patient education.

Variable	*f* (%)
Use of foot health services provided by chiropodist or podiatrist	
Yes	322 (59)
No	227 (41)
Frequency of visiting foot health services provided by chiropodist or podiatrist	
Once a month	14 (3)
Once in 3 months	74 (13)
Once in 6 months	60 (11)
Once a year	67 (12)
Rarely	123 (22)
Never	214 (38)
Satisfaction with foot health services provided by chiropodist or podiatrist	
Very satisfied	124 (29)
Satisfied	221 (52)
Not satisfied nor dissatisfied	71 (17)
Dissatisfied	6 (1)
Very dissatisfied	4 (1)
Has received patient education about foot health care	
Yes	211 (32)
No	447 (68)

Background questions were asked and included age (in years), duration of RA (years), gender, highest level of education, self‐perceived importance of foot health (1 = very important, 2 = important, 3 = somewhat important, 2 = slightly important 1 = not important), perceived impact of foot health on performing daily activities (5‐point scale from very much to very little 1 = very much, 2 = much, 3 = much or less, 4 = a little, 5 = very little) and a self‐rated level of current foot health (1 = poorest foot health, 10 = best possible foot health).

### 2.3 Data analysis

The data were analysed using descriptive statistics and thematic analysis. For descriptive statistics, frequencies, percentages, mean and range values were calculated using IBM SPSS Statistics for Windows (Version 29.0: IBM Corp). For open‐ended questions, the data consisted of 60 pages (Times New Roman, font 12 and spacing 1) and was analysed using thematic analysis [17]. First, all open‐ended responses were read several times to gain an initial understanding of the respondents' perceptions. Second, initial codes were generated by identifying phrases or sentences containing words related to the research questions (such as foot care, podiatry, services). This phase produced a total of 356 codes. Third, themes were formed by identifying common patterns in the codes, and the codes were combined into themes. Fourth, the themes were reviewed against the data set to ensure the accurate correspondence and representation of the data. This was done by comparing the identified themes with the data content. Fifth, the themes were named to reflect their content. The initial coding and forming of themes were conducted by one researcher (MS) with continuous discussion of the codes and category names together with the two other researchers in the team (SH, RS). The final themes and their content were confirmed within the research team.

### 2.4 Ethical considerations

The study obtained ethical approval from the University's ethical review board (ethical committee code: 35/2021). The study followed good scientific practice in every phase [18]. Permission to collect the data was obtained from the national patients' association according to their standard procedure. The patients' association named a contact person who sent the invitation to participate in the study to every potential participant identified from the member registry of the patients' association. The invitation consisted of the information letter, including a description of the study purpose, method of data collection, anonymity, confidentiality in data collection, analysis and reporting and the possibility to withdraw from the study at any point. In addition, the invitation had a link to the electronic survey. Before accessing and responding to the survey each participant gave their informed consent.

## 3 RESULTS

### 3.1 Description of participants

The mean age of the respondents was 60 years (SD 12.3, range 22–87) and the majority were female (*n* = 491, 90%). Most of the respondents had as their highest level of education in a university of applied sciences degree (*n* = 221, 40%) and vocational school degree (*n* = 147, 27%). On average, they have had RA for 15 years (SD 13.2, range 0–70). They perceived foot health as very important (*n* = 463, 84%). They believed their foot health affected their performance in daily activities very much (*n* = 230, 42%) or a lot (*n* = 213, 39%). The mean score for their self‐rated foot health was 6.7 (median 7) on a 10‐point scale (SD 1.8, range 1–10), indicating a rather good level of foot health (Table 1).

### 3.2 Use of and satisfaction with foot health services

About two thirds of the respondents (*n* = 322, 59%) had used foot health services provided by chiropodist or podiatrist because of foot problems. Those who visited foot health services reported having done so rarely (*n* = 123, 22%), once every third month (*n* = 74, 13%) or once a year (*n* = 67, 12%). They perceived the services being satisfied (*n* = 182, 52%). Regarding patient education about foot health delivered by health care professionals, most of the respondents (*n* = 383, 69%) reported having an insufficient amount of foot health information (Table 2).

### 3.3 Reasons for not using foot health services

**FIGURE 1:**
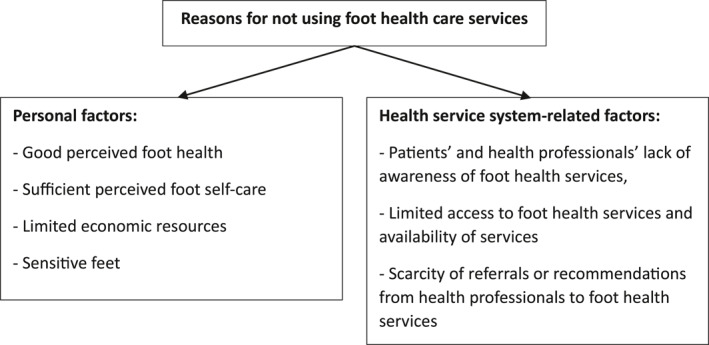
Respondents' (*n* = 565) perceived reasons for not using foot health services.

One third of the respondents (*n* = 214, 30%) reported not ever visiting foot health services. The reasons for not using foot health services consisted of two main themes: (1) personal factors, and (2) health service system‐related factors (Figure 1).

***Personal factors*** were good perceived foot health, sufficient foot self‐care, limited economic resources and sensitive feet.

Good perceived foot health was considered as a positive aspect and therefore external support for maintaining good foot health was not needed or considered.

…I have no foot problems…

(R3)

…I have no need for professional care as my feet are in excellent condition…

(R68)

Sufficient foot self‐care was regarded an important daily activity to maintain a good level of foot health. Respondents reported caring for their feet as well as possible, and therefore considered foot health services as redundant at this point. They reported that if their foot health should deteriorate, they would then seek professional help for their problems.

…I can take care of my own feet. So far it has been enough…

(R95)

…I can take care of my feet, I have no need for professional care…

(R21)

Limited economic resources was a major problem explaining why some respondents did not use foot health services. As the respondents considered that public foot health services were difficult to reach and access, therefore they considered private foot health care providers to help them with foot problems. Services provided by private podiatrists need to be paid for out of pockets by individual clients. The respondents perceived that the costs of private foot health care as too high, and they were not ready to pay for these services themselves. Instead, they decided to live with their feet without professional foot health care. The respondents also mentioned that they needed to prioritise their daily life activities and health choices before their foot health.

…I have no money. Private podiatry services are expensive.

(R203)

…I have small children, I need to buy them clothes, pay for food and electricity. I need to prioritise how I use my money.

(R284)

Sensitive feet were perceived as a hindrance when it came to using foot health services. Someone else touching their feet contributed to tickling and uncomfortable feelings.

…I do not like it when someone else touches my feet…

(R176)

…my feet are over‐sensitive; therefore, I cannot stay still if someone, even health professionals, touches my feet…

(R85)

***Health service system‐related factors*** were a patients' and health professionals' lack of awareness of foot health services, limited access to foot health services and the scarcity of referrals or recommendations from health professionals to foot health services.

A patients' and health professionals' lack of awareness of foot health services was described as a general unawareness of foot health services. The respondents reported having no understanding of foot health services, how those services could be accessed and how one would know who the correct service provider is. They also mentioned that they were unaware that foot health should be part of comprehensive care in overall care of RA. Moreover, the people with RA were unaware of why they should visit foot health services and how these services could benefit them. They mentioned that health professionals were not informing about foot care and podiatry services during their rheumatology or general practitioner appointments.

…I had no idea that my feet should be cared for.

(R4)

…I thought that having painful feet was part of living with rheumatoid arthritis…

(R83)

…I do not know how I can access foot care services…

(R115)

…I do not know if there is a chiropodist or podiatrist available in my living area, no information has provided to me…

(R478)

Limited access to foot health services was a major concern for respondents. In general, they perceived that there are not enough service providers in foot health and getting access to podiatry, for example, was difficult. They believed that public foot health services had decreased. The respondents were of the opinion that getting an appointment to podiatry required severe, painful and complicated foot problems. Minor foot problems, such as painless thickened toenails, corns of calluses or overlapping toes, were seen as less important and therefore often not seen as part of foot health services. The respondents also mentioned that the place where one lives also affects the availability of foot health services. They complained that not every city or rural area has its own chiropodist or podiatrist. Long distances between home and foot care provider limited respondents' willingness to travel to receive foot care.

…Foot care and podiatry services are not available…

(R370)

…People with diabetes receive foot care, but we rheumatoid arthritis patients do not. Our problems are not taken seriously…

(R165)

…The podiatrist in my city retired and nobody continued her work, so now we don’t have any podiatry services anymore…

(R601)

The scarcity of referrals or recommendations from health professionals to foot health services was a significant hindering factor. The respondents believed that health care professionals, such as general practitioners and rheumatologists, ignored their foot health. The respondents did not receive referrals to podiatry from health care professionals or general recommendations for care of their feet. This was seen as a major weakness. The respondents believed that health care professionals do not guide or educate them on how to self‐care for their feet or give advice on where to seek help if self‐care is not enough. Podiatry services in Finland are not compensated for by the Social Insurance Institution of Finland (KELA); whereas, many other services, such as physiotherapy, are. Therefore, respondents did not use podiatry services due to their high costs.

…No one in health care has recommended foot care to me…

(R134)

…I have never received a referral to podiatry, never…

(R275)

…I have mentioned my feet to my rheumatologist, but it has never led to anything…

(R541)

Previous unsatisfied experiences from public foot health services where foot health needs of people with RA were not met were reasons for not using services anymore. Respondents reported being dissatisfied with the received care. They mentioned that they themselves ended visits to foot care due to the poor care quality or dissatisfaction with the care results.

…After the foot care I have even more pain than before the appointment…

(R447)

…I got insoles, but those are not fitting to my shoes, waste of my time once you do not get what you need…

(R551)

### 3.4 Development areas for foot health services for people with RA

The respondents described many ideas for developing foot health services (Table 3). First, they highlighted that foot health services should be a part of comprehensive care for people with RA, the services should be easy to access and cost to client nothing or very little. The respondents strongly demanded that foot health services for people with RA should be at the same level as foot health services for people with diabetes mellitus.

…Podiatry should be integral part of care in RA…

(R5)

…Regular monitoring of foot health is important. I do not know if something is wrong in my feet, podiatrist knows…

(R17)

…Podiatry should be free of charge to everyone who has RA…

(R26)

…Podiatry should be at the same level as it is for people with diabetes. They can have referrals to podiatry, but we people with RA are left outside…

(R61)

Next, according to the respondents, a podiatrists should work in rheumatology clinics to promote multi‐professional collaboration and consultations during visits.

…I support multiprofessional care approach where rheumatologists, physiotherapists, podiatrists, nurses, all care my health needs, together…

(R32)

…A podiatrist should work in the rheumatology unit, it would be easy to seek help or consultation, I guess…

(R125)

Furthermore, the respondents considered an annual foot health check by a podiatrist as a key factor in promoting and monitoring foot health. An annual foot health check could provide not only follow‐up information about foot health but also serve as an opportunity to educate patients to care for their own feet. During a visit, footwear and insoles could also be evaluated and guidance on where to buy insoles or suitable footwear could be given. Regarding insoles and footwear, the respondents believed that those that are individually tailored or customised should come at a relatively low cost to clients. Lower costs, they believed, would encourage clients to buy and use such devices and thus health benefits would be achieved.

…Regular foot health assessment is important to know what is the condition of my feet…

(R201)

…I need help with insoles and footwear, during podiatric care I could get information also how to maintain and use insoles and orthoses…

(R48)

…Insoles cost too much, should be cheaper, then I would use them.

(R384)

Finally, the respondents regarded foot health‐related information as important. More foot health‐related guidance and education provided by a rheumatology nurse at a clinic or by a podiatrist through group education sessions or via the internet was greatly expected. Reliable information regarding foot self‐care was seen as important. The respondents also highlighted that other health care professionals working in rheumatology clinics or in occupational health care could benefit from having more information about foot health care to promote the comprehensive care for people with RA.

…I do not know how the care for my feet, I need support, education and practical advices…

(R104)

…They could organize group‐education, would be nice to meet other people with same health condition…

(R232)

…Everyone should receive patient education with no costs!…

(R203)

…Preventative aspects are important, not always the problems. I need information how to prevent problems occurring…

(R403)

…Information about foot self‐care to internet…

(R415)

**TABLE 3:** Respondents' (*n* = 565) perceptions of development areas in foot health care services for people with rheumatoid arthritis.

– Foot health care as part of comprehensive rheumatoid arthritis care – Easy and free of charge access to foot health care – Podiatrists in rheumatology clinics – Annual foot health checks – More foot health education during routine consultations

## 4 DISCUSSION

The perceptions of people with RA about foot health services are controversial. Those persons who have access to foot health services are satisfied with the services. However, there are many persons with RA who have no access to foot health care at all because of personal or health care organisational factors. It seems that access to foot health services are unequal even though the importance of foot health care has been stated in many international guidelines [3, 4]. People with RA identified many practical suggestions to help develop the content and delivery of foot health services.

The findings of this study build on the existing knowledge regarding the reasons [11] why people with RA do not use foot health services. In addition, this study demonstrates that people with RA experience unmet care needs and major health service system‐related obstacles in accessing foot health services. Client‐friendly and seamless foot health care paths for people with RA would support the timely access to care and respond to the foot health needs of people with RA [19]. The integration of podiatry services to rheumatology clinics could help improve access to foot health services and promote the monitoring of foot health in people with RA [20]. There is a growing need to increase the number of foot health professionals, such as podiatrists, who are dedicated to caring for foot problems in people with RA.

Patient education and general counselling regarding foot care for people with RA was seen as limited. Foot health education is a part of patient education and thus needs to be covered during regular appointments [21]. In future digital educational tools to support foot health and competence in foot self‐care among people with RA could be developed and tested [22, 23]. These kinds of digital tools could increase the awareness of foot health services and provide possibilities to update foot self‐care knowledge and skills among people with RA. Similarly, health care professionals could benefit from in‐service digitally implemented education about foot problems and foot care in people with RA. With this kind of continuous education, the competence of health care professionals to educate people with RA could be advanced and thus respond to patients' demands for foot‐related patient education at all levels of care.

Current foot health services seem to be deficient and many people with RA experience mild or severe foot problems that are untreated, thus limiting their ability to function in daily tasks. Some people with RA purchase foot health services from private practitioners. Access to public foot health services should be the priority in the comprehensive care chain for people with RA [8]. Equal access to foot health services could decrease the number of complicated foot problems which often impede functional ability. Having annual foot health checks, adequate foot self‐care education or care delivered by foot health professionals, the foot health of people with RA could be promoted and monitored, as recommended in international guidelines [3, 4]. Annual foot health services should be organised in a manner similar to that for patients with diabetes [24].

Future research could benefit from testing new models of care to ensure the timely and appropriate professional care of foot problems among people with RA. Health care systems could also benefit from restructuring the foot health services and include people with RA in the planning and testing of those services. By restructuring the system, health care professionals could find even more possibilities to refer the patient to, for example, podiatric care [25]. Patients would also be more satisfied with access to care as their waiting times would be shorter and they would see that their foot health is being taken into account. Co‐creation [26] together with people with RA could help find solutions to overcome the shortcomings of foot health care in the current health care systems. In future studies, the use of structured self‐rated foot health questionnaire together with instrument measuring perceived lower extremity function could provide a comprehensive overview of lower extremity health and thus produce evidence for the need of foot health services. Future research could also benefit from the big data approach by using a registry‐based study to inspect the number of referrals, number of visits to podiatric care and foot health status in people with RA.

Despite many faults and identified areas of development, those persons who have access to foot health services are grateful and believe they have quality care for their foot problems. In the future, there will be a continuing need to maintain the level of existing foot health services, but also to work towards equal access to services for all persons with RA. Patients with RA could benefit from nationally agreed standards and care chains that include individual podiatry plans based on comprehensive foot health assessments.

### 4.1 Limitations

There are some limitations that must be considered while interpreting the results. The survey data were collected electronically through a national patients' association. Selection bias might be present as only those who were members of the association and had access to a computer were able to participate in the study. Due to a relatively low response rate (24%, *n* = 565), the sample in this cross‐sectional study may not entirely represent all people with RA in Finland. However, the study was on national level representative of a wide age range of respondents. The results provide a general description of participants' perceptions; however, wider generalisation of the results is warranted. The data were collected using a questionnaire which was developed for the purposes of this study. Multiple‐scaled structured and open‐ended questions were constructed on the basis of previous research [9, 27] and expertise of the research team. Lack of patient involvement in the questionnaire development may be considered as limitation. As the questions do not form any scale, due to variation in response options, no testing of psychometric properties was conducted. The questionnaire was pilot tested with 10 persons with RA to ensure the technical functionality of the online survey. The pilot test led to no changes in the online survey.

The trustworthiness of the study was evaluated through credibility, transferability, dependability, confirmability and reflexivity [28]. To increase the credibility of the study, parts of the preliminary results were shared within the research team and peer debriefing was used to test the findings and interpretations within the research team. Limitation relating the trustworthiness of this study is the lack of respondent validation (or member checking) as it was not possible conduct due to anonymous nature of the survey. Transferability of the findings was supported by describing the study setting, context and results in‐detail which could allow the transfer of findings to other contexts or settings with other respondents. To support dependability, the study process was reported in detail to make it possible for any future researchers to repeat the study. To increase confirmability, the findings were reported as transparently and justified as possible. Reflexivity was highlighted through the research process. The data analysis was conducted primarily by one researcher (MS). Reflexive notes by MS were kept during the analysis process to record the personal reflections and decisions during the research process. Two researchers in the research team have degree in podiatry (MS, SH), which may have influenced the interpretation of the results. However, having one researcher (RS) with expertise in health services research have confirmed the results and interplay between results and implications for practice.

## 5 CONCLUSIONS

Foot health services are seen as an important part of the overall care of people with RA. However, the study findings indicate that there are unmet foot health needs. Dedicated foot health care services are limited and people with RA do not have equal access to these services. The main reasons for not seeking foot health services are personal factors, such as financial hardship, or health service system‐related factors, such as a lack of awareness of foot health services and their provision. Seamless care chains for people with RA, including multi‐professional collaboration, could support the functional health and well‐being of these patients. In the future, people with RA would benefit from the integration of podiatry services within rheumatology clinics and the development of new models of care delivery.

## AUTHOR CONTRIBUTIONS

**Minna Stolt**: Conceptualization; data curation; formal analysis; funding acquisition; investigation; project administration; writing – original draft; writing – review & editing. **Sasu Hyytiä**: Conceptualization; formal analysis; writing – review & editing. **Riitta Suhonen**: Conceptualization; methodology; supervision; writing – review & editing.

## CONFLICT OF INTEREST STATEMENT

The authors declare that they have no competing interests.

## ETHICS STATEMENT

The study obtained ethical approval from the University's ethical review board (ethical committee code: 35/2021). Before accessing and responding to the survey each participant gave their informed consent.

## CONSENT FOR PUBLICATION

Not applicable.

## Data Availability

The datasets used and/or analysed during the current study are available from the corresponding author on reasonable request.

## References

[1] Almutairi, K. , J. Nossent , D. Preen , H. Keen , and C. Inderjeeth . 2021. “The Global Prevalence of Rheumatoid Arthritis: A Meta‐Analysis Based on a Systematic Review.” Rheumatology International 41(5): 863–877. 10.1007/s00296-020-04731-0. 33175207

[2] Björk, M. , I. Thyberg , E. Valtersson , G. Östlund , B. Stenström , and A. Sverker . 2018. “Foot Barriers in Patients with Early Rheumatoid Arthritis: An Interview Study Among Swedish Women and Men.” Arthritis Care & Research 70(9): 1348–1354. 10.1002/acr.23486. 29195001

[3] Combe, B. , R. Landewe , C. I. Daien , C. Hua , D. Aletaha , J. M. Álvaro‐Gracia , M. Bakkers , et al. 2017. “2016 Update of the EULAR Recommendations for the Management of Early Arthritis.” Annals of the Rheumatic Diseases 76(6): 948–959. 10.1136/annrheumdis-2016-210602. 27979873

[4] Tenten‐Diepenmaat, M. , M. van der Leeden , T. P. M. Vliet Vlieland , J. Dekker , and RA Foot Expert Group . 2018. “Multidisciplinary Recommendations for Diagnosis and Treatment of Foot Problems in People with Rheumatoid Arthritis.” Journal of Foot and Ankle Research 11(1): 37. 10.1186/s13047-018-0276-z. 29988776 PMC6030746

[5] Williams, A. E. , and A. S. Graham . 2012. “My Feet: Visible, but Ignored…' A Qualitative Study of Foot Care for People with Rheumatoid Arthritis.” Clinical Rehabilitation 26(10): 952–959. 10.1177/0269215511434995. 22275462

[6] Tehan, P. E. , T. Morpeth , A. E. Williams , N. Dalbeth , and K. Rome . 2019. ““Come and Live with My Feet and You'll Understand" ‐ A Qualitative Study Exploring the Experiences of Retail Footwear in Women with Rheumatoid Arthritis.” Journal of Foot and Ankle Research 12(1): 15. 10.1186/s13047-019-0328-z. 30911335 PMC6416983

[7] de Souza, S. , R. Williams , and H. Lempp . 2016. “Patient and Clinician Views on the Quality of Foot Health Care for Rheumatoid Arthritis Outpatients: A Mixed Methods Service Evaluation.” Journal of Foot and Ankle Research 9: 1. 10.1186/s13047-015-0133-2. 26740821 PMC4702354

[8] Wilson, O. , J. Kirwan , E. Dures , E. Quest , and S. Hewlett . 2017. “The Experience of Foot Problems and Decisions to Access Foot Care in Patients with Rheumatoid Arthritis: A Qualitative Study.” Journal of Foot and Ankle Research 10(1): 4. 10.1186/s13047-017-0188-3. 28138340 PMC5264322

[9] Laitinen, A. M. , C. Boström , S. Hyytiä , and M. Stolt . 2022. “Experiences of Foot Health in Patients with Rheumatoid Arthritis: A Qualitative Study.” Disability & Rehabilitation 44(1): 88–95. 10.1080/09638288.2020.1758966. 32352848

[10] Blake, A. , P. J. Mandy , and G. Stew . 2013. “Factors Influencing the Patient with Rheumatoid Arthritis in Their Decision to Seek Podiatry.” Musculoskeletal Care 11(4): 218–228. 10.1002/msc.1044. 23348757

[11] Hendry, G. J. , K. A. Gibson , K. Pile , L. Taylor , V. Du Toit , J. Burns , and K. Rome . 2013. ““They Just Scraped off the Calluses”: A Mixed Methods Exploration of Foot Care Access and Provision for People with Rheumatoid Arthritis in South‐Western Sydney, Australia.” Journal of Foot and Ankle Research 6(1): 34. 10.1186/1757-1146-6-34. 23938103 PMC3751079

[12] McCulloch, L. , A. Borthwick , A. Redmond , K. Edwards , R. Pinedo‐Villanueva , D. Prieto‐Alhambra , A. Judge , N. K. Arden , and C. J. Bowen . 2018. “UK Podiatrists' Experiences of Podiatry Services for People Living with Arthritis: A Qualitative Investigation.” Journal of Foot and Ankle Research 11(1): 27. 10.1186/s13047-018-0262-5. 29928316 PMC5989380

[13] Paterson, K. L. , C. Harrison , H. Britt , R. S. Hinman , and K. L. Bennell . 2018. “Management of Foot/ankle Osteoarthritis by Australian General Practitioners: An Analysis of National Patient‐Encounter Records.” Osteoarthritis and Cartilage 26(7): 888–894. 10.1016/j.joca.2018.03.013. 29656142

[14] Nguyen, V. , A. Brenton‐Rule , N. Dalbeth , K. Rome , and S. Stewart . 2022. “An Evaluation of Podiatry Service Use for People with Inflammatory Rheumatic Diseases: A Review of a Rheumatology Podiatry Clinic in Aotearoa New Zealand.” Journal of Foot and Ankle Research 15(1): 36. 10.1186/s13047-022-00542-7. 35578311 PMC9108704

[15] Hennessy, K. , A. Williams , M. Steultjens , and J. Woodburn . 2013. “Podiatry Care in Rheumatoid Arthritis: Differences between Current and Ideal Service Provision.” Journal of Foot and Ankle Research 6(Suppl 1): O16. 10.1186/1757-1146-6-s1-o16.

[16] Rheumatoid arthritis. Current Care Guidelines . 2022. Working Group Set up by the Finnish Medical Society Duodecim and the Finnish Rheumatology Society. Helsinki: The Finnish Medical Society Duodecim. www.kaypahoito.fi. Accessed 31st Aug 2023.

[17] Braun, V. , and V. Clarke . 2012. Thematic Analysis. Washington: American Psychological Association.

[18] ALLEA . 2023. The European Code of Conduct for Research Integrity – Revised Edition. https://allea.org/code‐of‐conduct/. Accessed 9th Oct 2023.

[19] Carter, K. , P. P. Cheung , K. Rome , A. Santosa , and M. Lahiri . 2017. “Increasing Podiatry Referrals for Patients with Inflammatory Arthritis at a Tertiary Hospital in Singapore: A Quality Improvement Project.” The Foot 31: 6–12. 10.1016/j.foot.2016.12.002. 28282539

[20] Dando, C. , D. Bacon , A. Borthwick , and C. Bowen . 2020. “Stakeholder Views of Podiatry Services in the UK for People Living with Arthritis: A Qualitative Study.” Journal of Foot and Ankle Research 13(1): 58. 10.1186/s13047-020-00427-7. 32972443 PMC7517686

[21] Graham, A. S. , and A. E. Williams . 2016. “Foot Health Education for People with Rheumatoid Arthritis: '…. A Game of Chance…' ‐ A Survey of Patients' Experiences.” Musculoskeletal Care 14(1): 37–46. 10.1002/msc.1111. 26076891

[22] Nikiphorou, E. , E. J. F. Santos , A. Marques , P. Böhm , J. W. Bijlsma , C. I. Daien , B. A. Esbensen , et al. 2021. “2021 EULAR Recommendations for the Implementation of Self‐Management Strategies in Patients with Inflammatory Arthritis.” Annals of the Rheumatic Diseases 80(10): 1278–1285. 10.1136/annrheumdis-2021-220249. 33962964 PMC8458093

[23] Knudsen, L. R. , K. Lomborg , E. M. Hauge , H. A. Zangi , and A. de Thurah . 2023. “The WebRA Study: Opportunities and Challenges in Digital Patient Education from the Perspective of Patients with Rheumatoid Arthritis: A Qualitative Study.” Patient Education and Counseling 116: 107969. 10.1016/j.pec.2023.107969. 37672918

[24] Schaper, N. C. , J. J. van Netten , J. Apelqvist , S. A. Bus , R. Fitridge , F. Game , M. Monteiro‐Soares , E. Senneville , and IWGDF Editorial Board . 2023. “Practical Guidelines on the Prevention and Management of Diabetes‐Related Foot Disease (IWGDF 2023 Update).” Diabetes/Metabolism Research and Reviews 40(3): e3657. 10.1002/dmrr.3657. 37243927

[25] Chapman, L. S. , M. Backhouse , L. Bearne , L. Cherry , G. Cleary , J. Davey , R. Ferguson , et al. 2022. “Management of Foot Health in People with Inflammatory Arthritis: British Society for Rheumatology Guideline Scope.” Rheumatology 61(10): 3907–3911. 10.1093/rheumatology/keac340. 35772746 PMC9536780

[26] Amorim, J. , and A. C. Ventura . 2023. “Co‐Created Decision‐Making: From Co‐production to Value Co‐Creation in Health Care.” The Journal of Medicine Access 7: 27550834231177503. 10.1177/27550834231177503. 37323851 PMC10262615

[27] Stolt, M. , R. Suhonen , and H. Leino‐Kilpi . 2017. “Foot Health in Patients with Rheumatoid Arthritis‐A Scoping Review.” Rheumatology International 37(9): 1413–1422. 10.1007/s00296-017-3699-0. 28324133

[28] Nowell, L. S. , J. M. Norris , D. E. White , and N. J. Moules . 2017. “Thematic Analysis: Striving to Meet the Trustworthiness Criteria.” International Journal of Qualitative Methods 16: 1–13. 10.1177/1609406917733847.

